# Experimental and Modelling Analysis of the Hyperthermia Properties of Iron Oxide Nanocubes

**DOI:** 10.3390/nano11092179

**Published:** 2021-08-25

**Authors:** Riccardo Ferrero, Gabriele Barrera, Federica Celegato, Marta Vicentini, Hüseyin Sözeri, Nuray Yıldız, Ceren Atila Dinçer, Marco Coïsson, Alessandra Manzin, Paola Tiberto

**Affiliations:** 1 Istituto Nazionale di Ricerca Metrologica (INRIM), 10135 Torino, Italy; f.celegato@inrim.it (F.C.); m.vicentini@inrim.it (M.V.); m.coisson@inrim.it (M.C.); a.manzin@inrim.it (A.M.); p.tiberto@inrim.it (P.T.); 2 Politecnico di Torino, 10129 Torino, Italy; 3 TÜBİTAK Ulusal Metroloji Enstitüsü (UME), 41470 Gebze, Kocaeli, Turkey; huseyin.sozeri@tubitak.gov.tr; 4 Department of Chemical Engineering, Ankara University, Tandoğan, 06100 Ankara, Turkey; nyildiz@eng.ankara.edu.tr (N.Y.); catila@eng.ankara.edu.tr (C.A.D.)

**Keywords:** nanomedicine, magnetic hyperthermia, magnetic nanoparticles, iron oxide nanocubes, chemical synthesis, magnetometry, thermometric measurements, micromagnetic simulations, thermal simulations

## Abstract

The ability of magnetic nanoparticles (MNPs) to transform electromagnetic energy into heat is widely exploited in well-known thermal cancer therapies, such as magnetic hyperthermia, which proves useful in enhancing the radio- and chemo-sensitivity of human tumor cells. Since the heat release is ruled by the complex magnetic behavior of MNPs, a careful investigation is needed to understand the role of their intrinsic (composition, size and shape) and collective (aggregation state) properties. Here, the influence of geometrical parameters and aggregation on the specific loss power (SLP) is analyzed through in-depth structural, morphological, magnetic and thermometric characterizations supported by micromagnetic and heat transfer simulations. To this aim, different samples of cubic Fe_3_O_4_ NPs with an average size between 15 nm and 160 nm are prepared via hydrothermal route. For the analyzed samples, the magnetic behavior and heating properties result to be basically determined by the magnetic single- or multi-domain configuration and by the competition between magnetocrystalline and shape anisotropies. This is clarified by micromagnetic simulations, which enable us to also elucidate the role of magnetostatic interactions associated with locally strong aggregation.

## 1. Introduction

In the last decade, the interest towards the application of magnetic nanoparticles (MNPs) in biomedicine has increased exponentially, being employed in diagnostics, as contrast agents in magnetic resonance imaging (MRI) or tracers in magnetic particle imaging (MPI), as well as in therapeutics, as heat mediators in heat-assisted drug release and magnetic hyperthermia [1,2,3]. Hyperthermia is an oncological therapy that can be used to sensitize tumor cells to radiotherapy and chemotherapy, allowing the reduction of radiation and drug dose with the consequent limitation of the related side effects. Magnetic hyperthermia exploits the capability of MNPs to generate heat when exposed to an alternating current (AC) magnetic field with frequency in the range 50 kHz–1 MHz. As an example, Fe_3_O_4_ (magnetite) NPs have been successfully tested in many clinical trials, using magnetic fields with frequency of 100 kHz and amplitude variable between 2.5 kA/m and 18 kA/m [4,5,6].

The hyperthermia efficacy of MNPs is usually measured by means of the specific loss power (SLP), also known as specific absorption rate (SAR), which expresses the power dissipated per unit mass of magnetic material [7,8]. The mechanisms contributing to SLP involve the alignment of the MNP magnetic moment along the direction of the AC magnetic field by the rotation of the magnetization vectors within MNPs (hysteresis and Néel relaxation) or by the mechanical rotation of the MNPs in the medium (Brownian relaxation) [9,10]. The latter is typically inhibited in the tumor microenvironment [11]. The magnetic behavior and the consequent heating properties are strongly influenced by MNP size, with the presence of two critical dimensions, which are material dependent [2,12]. The lowest critical size is associated with the transition from superparamagnetic to single-domain ferromagnetic behavior, above which the coercivity (zero for the superparamagnetic state), and thus, the hysteresis losses gradually increase reaching a maximum. This occurs in correspondence of the highest critical size, which corresponds to the transition to multi-domain ferromagnetic behavior, after which coercivity and hysteresis losses start to decrease with dimension.

Large research efforts have been devoted to the engineering of novel MNPs with high SLP values, through the increase in hysteresis losses. Beside size [13], another parameter that can be varied to tune the hysteresis contribution is the shape [14,15,16,17], for example, via the introduction of faceting and elongation [18]. To this aim, Fe_3_O_4_ NPs with different geometries (e.g., rods [19], disks [20], rings [21,22], octahedrons [23,24], and cubes [25,26,27]) have been investigated for potential application in magnetic hyperthermia. Thanks to the introduction of even weak shape anisotropy, increments in the SLP values can be obtained when moving from spherical to cubic Fe_3_O_4_ NPs of certain dimension, as already demonstrated by calorimetric measurements in water and agar media [25,26]. For instance, good heating efficacy of Fe_3_O_4_ nanocubes with size around 20 nm was found in both in vitro [27] and in vivo studies when treating epidermoid carcinoma xenografts in mice [28]. Fe_3_O_4_ nanocubes were also tested as drug carriers, in order to obtain therapeutic agents with dual function, combining magnetic hyperthermia and heat-mediated chemotherapy [29].

Another parameter that can influence the hysteresis losses and thus the SLP values of MNPs is the magnetostatic interaction strength, which strongly depends on the aggregation state (e.g., chain, conglomerate). Several studies investigated the possibility of exploiting the arrangement of MNPs at the nanoscale to improve their hyperthermia properties [30,31,32,33,34,35]. As an example, it was demonstrated that the shape anisotropy induced by chain formation leads to an enhancement of heating efficiency by up to a factor of two in the case of 40 nm Fe_3_O_4_ NPs [35]. One more way to modify the SLP values is acting directly on the chemical composition of MNPs, e.g., doping iron oxides NPs with different metals (Co, Ni, Zn, Cu, Gd, Mn) [36,37]. The doped ferrites can show high magnetocrystalline anisotropy, resulting in larger coercivity and thus greater hysteresis losses.

The presence of many influencing parameters (material features, size, shape, aggregation state) makes complex to distinguish and quantify their contribution to the heating performance, requiring before in vitro and in vivo tests an exhaustive experimental and modelling analysis of MNP magnetic and calorimetric properties. To this aim, here we present a detailed characterization of Fe_3_O_4_ NPs with cubic shape, produced via hydrothermal route by varying synthesis parameters (temperature and reaction time). Four samples were prepared, with average size between 15 nm and 160 nm, and magnetic behavior that mainly falls in the single-domain ferromagnetic regime or in the multi-domain one, as demonstrated by magnetometric measurements combined with micromagnetic calculations of the static hysteresis loops. The micromagnetic simulations were conducted first on single MNPs of variable size to determine the second critical size, where the transition from single- to multi-domain behavior occurs. Then, they were carried out on MNP assemblies in the form of chains and clusters to investigate the possible contribution of magnetostatic interactions to remanence, coercivity and specific energy losses.

Finally, the heating efficiency was characterized by performing thermometric measurements of the MNPs dispersed in water as well as thermal modelling. In particular, the thermometric characterization was carried out under uniform AC magnetic fields with peak amplitude *Ĥ*_a_ and frequency *f* that fall between the limits of Atkinson–Brezovich (*Ĥ*_a_·*f* ≤ 4.85 · 10^8^ Am^–1^s^–1^) [38] and Hertz–Dutz (*Ĥ*_a_·*f* ≤ 5 · 10^9^ Am^−1^s^−1^) [39]. This setting of the AC magnetic field parameters might assure that eddy current effects are maintained below safe and tolerable limits. It is worth noting that among the first clinical trials of magnetic hyperthermia, good tolerability was documented when exposing patients with different types of tumors to a magnetic field of 100 kHz, with amplitude variable up to 18 kA/m [4,5,6]. Anyway, caution has to be taken when planning a treatment, to avoid the occurrence of hot spots and overheating of healthy tissues [40].

## 2. Materials and Methods

### 2.1. Magnetic Nanoparticle Synthesis

Fe_3_O_4_ NPs were synthesized by hydrothermal method [41]. The molar ratio of Fe^2+^/Fe^3+^ was adjusted to 1:2, and the iron salt solution was prepared by using deoxygenated deionized water. Afterward, the solution was transferred into a polytetrafluoroethylene lined autoclave. Then, 1 M aqueous NaOH solution was dropped into the iron oxide solution under nitrogen gas. The autoclave was operated at different temperatures between 150 °C and 200 °C, with different reaction times (12 h and 24 h). Four samples were produced (namely, #1, #2, #3, and #4); sample labelled as #1 was heat treated at 150 °C for 12 h, #2 at 150 °C for 24 h, #3 at 175 °C for 12 h and #4 at 200 °C for 24 h. The prepared Fe_3_O_4_ NPs were collected magnetically and then washed with deionized water three times and dried at 60 °C under vacuum for 24 h.

Iron (II) chloride tetrahydrate (FeCl_2_·4H_2_O, ≥99%), iron (III) chloride hexahydrate (FeCl_3_·6H_2_O, ≥98%) and sodium hydroxide (NaOH, ≥98%) employed in the synthesis process were purchased from Sigma-Aldrich^®^ (St. Louis, MO, USA). All products were used as received without any further purification. 

Fe_3_O_4_ NPs were formed during the following chemical reaction:

(1) $${\mathrm{Fe}}^{2+}{+2\mathrm{Fe}}^{3+}{+8\mathrm{OH}}^{–}\to {\mathrm{Fe}}_{3}O_{4}{+4H}_{2}O$$

### 2.2. Structural, Morphological and Dimensional Characterization

The investigation of the crystal phase of the synthesized MNPs was carried out by X-ray Diffractometry (XRD) [42], employing the instrument Shimadzu XRD-6000 (Kyoto, Japan); the obtained spectra were refined using the Rietveld method.

Transmission Electron Microscopy (TEM) [42] was performed to determine the morphology and size distribution of the MNPs in each of the four samples. The TEM images, obtained with the instrument JEOL JEM-2100 HRTEM (Tokyo, Japan), were analyzed by using the open-source ImageJ software [43].

### 2.3. Magnetometric Characterization

The magnetic characterization was performed at room temperature by means of Vibrating Sample Magnetometry (VSM) [44], using the instrument Lakeshore, Model 7410 (Westerville, OH, USA). The measurement of the hysteresis loops was carried out under direct current (DC) magnetic fields, varied from −1200 kA/m to 1200 kA/m in steps of 200 A/m.

### 2.4. Thermometric Characterization

Thermometric measurements were performed by means of a custom-built setup described in detail elsewhere [45]. A uniform AC magnetic field with a frequency *f* of 100 kHz and a peak amplitude *Ĥ*_a_ in the range 24–48 kA/m was applied to an aqueous suspension of Fe_3_O_4_ NPs at a known concentration. The magnetic field parameters were selected to satisfy the Hergt–Dutz criterion [39].

A fiber optic thermometer records the temperature increase in the magnetic solution induced by the power released by the MNPs and the subsequent cooling to room temperature after the magnetic field is turned off. The experimental curves were analyzed by an ad hoc thermodynamic analytical model, in order to obtain a direct estimation of the SLP values [45]. This model takes into account the parasitic heating of the water and the heat exchange with the surrounding environment under non-adiabatic conditions, which can be limited by inserting the sample within a thermal insulating polystyrene foam tube [8].

### 2.5. Micromagnetic Simulations

The static hysteresis loops of the Fe_3_O_4_ NPs were evaluated by means of an in-house 3D micromagnetic code [46], which solves the Landau–Lifshitz–Gilbert (LLG) equation:

(2) $$\frac{\partial M}{\partial t}=-\frac{\gamma }{1+\alpha^{2}}M\times {\left[H_{\mathrm{eff}}+\frac{\alpha }{M_{s}}\left(M\times H_{\mathrm{eff}}\right)\right]}_{,}$$

where **M** is the magnetization vector of constant amplitude equal to saturation magnetization *M*_s_; *γ* = 2.21 · 10^5^ m A^−1^ s^−1^ is the absolute value of the gyromagnetic ratio, and *α* is the damping coefficient [47]. The effective field **H**_eff_ is the sum of the applied field **H**_a_, the magnetostatic field **H**_m_, the exchange field **H**_ex_, the magnetocrystalline anisotropy field **H**_an_ and the thermal field **H**_th_ [47].

The magnetostatic field is expressed as

(3) $$H_{m}=-\frac{1}{4\pi }\nabla \overset{\;}{\underset{\Omega }{\int }}\nabla^{\prime }\left(\frac{1}{\left|r-r^{\prime }\right|}\right)\cdot M\left(r^{\prime }\right)d^{3}{r^{\prime }}_{,}$$

where **r** is the vector position of the calculation point, and **r’** is the integration variable.

The exchange field is expressed as

(4) $$H_{\mathrm{ex}}=\frac{2k_{\mathrm{ex}}}{\;\mu_{0}M_{s}^{2}}\nabla^{2}M_{,}$$

where µ_0_ = 4π × 10^−7^ H/m is the magnetic permeability of vacuum, and *k*_ex_ is the exchange constant.

For the case of cubic anisotropy with crystal axes coinciding with the coordinate ones, the magnetocrystalline anisotropy field is expressed as

(5) $$H_{\mathrm{an}}=-\frac{2Κ}{\;\mu_{0}M_{s}}m_{,}$$

where K is the magnetic anisotropy tensor, defined as

(6) $$K=\left[\begin{array}{ccc} K_{1}\left(m_{y}^{2}+m_{z}^{2}\right)+K_{2}m_{y}^{2}m_{z}^{2} & 0 & 0\\ 0 & K_{1}\left(m_{x}^{2}+m_{z}^{2}\right)+K_{2}m_{x}^{2}m_{z}^{2} & 0\\ 0 & 0 & K_{1}\left(m_{x}^{2}+m_{y}^{2}\right)+K_{2}m_{x}^{2}m_{y}^{2} \end{array}\right]$$

with **m** being the normalized magnetization vector, and *K*_1_ and *K*_2_, the first and second order cubic anisotropy constants, respectively.

When included, the contribution from **H**_th_ is calculated following the Langevin approach and the fluctuation–dissipation theorem [48], resulting in:

(7) $$H_{\mathrm{th}}=\eta \left(r,t\right)\sqrt{\frac{2\alpha k_{B}T}{\gamma \mu_{0}M_{s}\Delta s^{3}\Delta t}},$$

where *T* is the absolute temperature, and *k*_B_ is the Boltzmann constant. The components of stochastic vector **η** are Gaussian random numbers, uncorrelated in space and time, and with zero mean value and dispersion 1. Parameter Δ*s* is the average size of the grid introduced for the spatial discretization and Δ*t* is the time-step [49].

The 3D micromagnetic solver, described in detail in [46], uses a spatial discretization based on a grid of hexahedral cells, where **M** and **H**_eff_ are assumed to be uniform. GPU-parallelization is exploited to speed up the computation. The spatial integration of **H**_m_ is performed with a Fast Fourier Transform (FFT) algorithm, implementing the tensor approach [50] and calculating the resulting Green’s surface integrals with 36 × 36 quadrature nodes; **H**_ex_ is computed with a 26-node-based finite-difference technique [51]. The time integration is carried out by means of a geometric integration method based on the Cayley transform, in order to preserve the constraint on the magnetization amplitude [52,53]. Its time-adaptive variant is used, fixing the truncation error to 10^−5^ [46].

In all the micromagnetic simulations, the properties of Fe_3_O_4_ were set as follows: saturation magnetization *M*_s_ = 410 kA/m; exchange constant *k*_ex_ = 12 pJ/m; cubic magnetocrystalline anisotropy with first anisotropy constant *K*_1_ = −13.5 kJ/m^3^ and second anisotropy constant *K*_2_ = −4.4 kJ/m^3^_._ To accelerate the computation of equilibrium points along the static hysteresis loop and use larger time-steps, the damping coefficient *α* was fixed to 0.1 when the thermal field is not included [53,54], employing the procedure detailed in [53]; otherwise, it was fixed to 0.02. The cell size chosen for spatial discretization was varied between 1.25 nm and 4.1 nm, accordingly to the MNP dimension.

### 2.6. Thermal Simulations

To support thermometric measurements, we carried out thermal simulations by means of an in-house 3D finite element code, which solves the heat transfer equation under the hypothesis of negligible convection phenomena [55]. This results in

(8) $$\rho C_{p}\frac{\partial T}{\partial t}=\nabla \cdot k\nabla T+Q_{\mathrm{MNPs}}+Q_{\mathrm{ext}},$$

where *T* is the temperature, *ρ* is the mass density, *C*_p_ is the heat capacity, *k* is the thermal conductivity, *Q*_MNPs_ is the heating power per unit volume produced by the MNPs in the particle-fluid suspension (MNPs plus water), and *Q*_ext_ is the heating power per unit volume due to external field sources. 

In particular, *Q*_MNPs_ is defined as

(9) $$Q_{\mathrm{MNPs}}=\mathrm{SLP}\cdot m_{\mathrm{MNPs}}/V_{\mathrm{water}},$$

where SLP is the value of the specific loss power of MNPs estimated from the thermometric measurements; *m*_MNPs_ is the mass of MNPs within the particle-fluid suspension, where MNPs are assumed to be uniformly dispersed, and *V*_water_ is the water volume [8].

*Q*_ext_ takes into account parasitic eddy current heating effects, which can occur in the aqueous suspension under the exposure to the AC magnetic field. Here, it is not numerically evaluated as in [56], but it is obtained along the calibration of the experimental set-up, by means of the thermodynamic analytical model detailed in [45]. In particular, *Q*_ext_ is determined by fitting the results of preliminary thermometric measurements performed on a sample of water with volume *V*_water_, before the addition of MNPs.

Equation (8) is completed by the following boundary condition:

(10) $$q=-k\nabla T\cdot n=-h{\left(T_{\mathrm{ext}}-T\right)}_{,}$$

where *q* is the outward heat flux, **n** is the outward normal vector to the boundary surface, *h* is the heat transfer coefficient, which includes exterior convective cooling effects, and *T*_ext_ is the external temperature that can be variable in time. The initial temperature (i.e., at time instant *t* = 0) is fixed to *T*_ext_.

The solution of Equation (8) was obtained by using a tetrahedral mesh and by approximating *T* with linear shape functions; the time integration was performed with Crank–Nicholson’s method [55].

## 3. Results and Discussion

This section deals with the characterization of the produced Fe_3_O_4_ NPs. The aim is to determine structural properties via XRD analysis, dimensional properties (MNP size and shape) via TEM imaging, magnetic properties (remanent magnetization, coercivity, hysteresis loop) via VSM measurements and hyperthermia properties via thermometric measurements. Micromagnetic and thermal modelling supports the experimental analysis, providing a physical insight of the obtained results.

### 3.1. Structural, Morphological and Dimensional Properties

**Figure 1:**
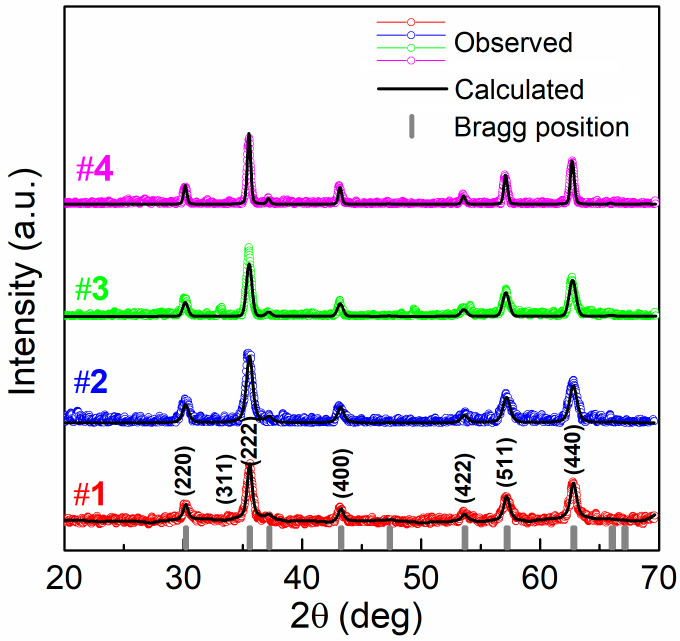
X-ray Diffractometry (XRD) spectra of the four synthetized samples of Fe_3_O_4_ nanoparticles (NPs), analyzed by the Rietveld refinement method.

The XRD patterns of the synthesized MNPs are shown in Figure 1. All spectra reveal a crystalline phase with diffraction peaks indexed with the cubic spinel structure of magnetite (Fe_3_O_4_) with space group of $Fd\;\bar{3}m$ (JCPDS card No. 19-0629). The lattice parameter *a* = *b* = *c* of the cubic cell, calculated using TREOR90 software [57], varies between 8.363 Å and 8.381 Å (see Table 1) and is close to the standard lattice parameter of bulk magnetite (8.393 Å, JCPDS card No. 19-0629).

**Figure 2:**
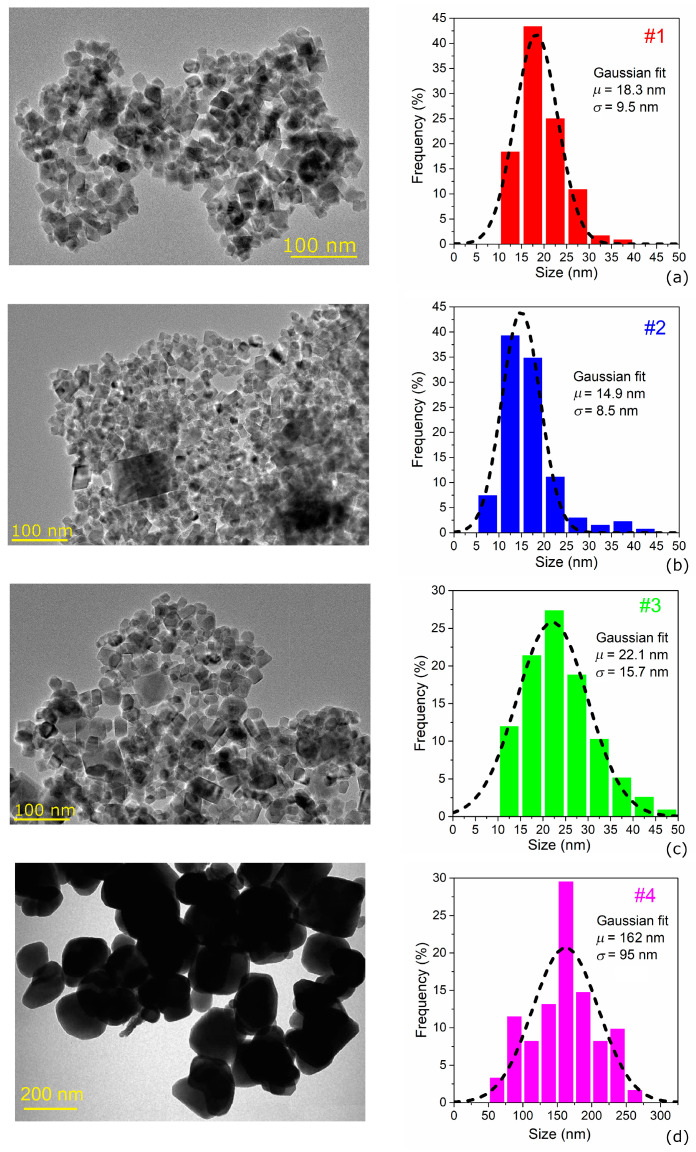
Transmission Electron Microscopy (TEM) images of the four samples of Fe_3_O_4_ NPs, with relative size histograms fitted by a Gaussian function having mean value *µ* and standard deviation *σ*. The mean size is equal to: (**a**) 18.3 nm for sample #1; (**b**) 14.9 nm for sample #2; (**c**) 22.1 nm for sample #3; (**d**) 162 nm for sample #4.

Representative TEM images and size distributions for each of the four samples are shown in the left and right panels of Figure 2, respectively. The TEM images reveal a well-defined cubic morphology of the Fe_3_O_4_ NPs with a different edge size as a function of synthesis parameters. Samples #1 and #2 are characterized by a narrow MNP size distribution well fitted by a Gaussian function having mean value (*μ*) of 18.3 nm and 14.9 nm and standard deviation *(σ*) of 9.5 nm and 8.5 nm, respectively. In these samples, a large fraction of MNPs has dimensions between 10 nm and 20 nm, range to which the critical size for transition from superparamagnetism to ferromagnetism of Fe_3_O_4_ NPs is expected to belong [58,59].

The MNP size distribution related to sample #3 spans a wider range at higher values; in particular, the corresponding Gaussian fit curve results in *μ* = 22.1 nm and *σ* = 15.7 nm. No MNPs larger than 50 nm are found in samples #1–3. In contrast, the MNPs in sample #4, due to the higher temperature (200 °C) and longer time (24 h) of reaction synthesis, exhibit very large sizes with an almost uniform distribution in the wide 50–275 nm range and only one higher bar centered at 162 nm. In this case, only a rough Gaussian fit can be extracted with *µ* = 162 nm and *σ* = 95 nm.

### 3.2. Hysteresis Loop Measurement

**Figure 3:**
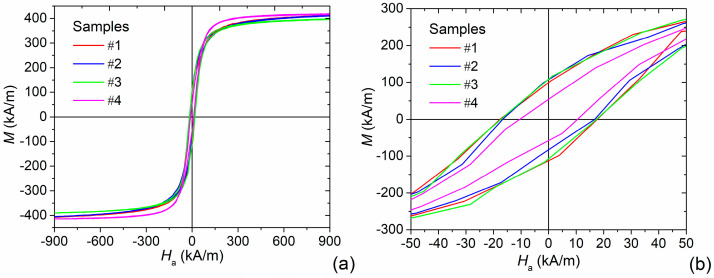
(**a**) Static hysteresis loops of the four samples of Fe_3_O_4_ NPs, measured via Vibrating Sample Magnetometry (VSM); (**b**) magnification of the central part of the loops to highlight remanence and coercivity.

The room-temperature static hysteresis loops of all the samples are shown in Figure 3a; details of the same curves in a narrow field interval around zero are magnified in Figure 3b. All curves exhibit a typical magnetic hysteretic behavior resulting from the ferrimagnetic ordering of Fe_3_O_4_ spinel structure and from the magnetic blocked state of a large fraction of MNPs in each sample, as predicted by the size distributions in Figure 2. The values of saturation magnetization (*M*_s_), remanent magnetization (*M*_r_) and coercivity (*H*_c_) are listed in Table 1. *M*_s_ was estimated by fitting the high field portion of the hysteresis loop with the following expression:

(11) $$M=M_{s}\left(1-\frac{\delta }{H_{a}}-\frac{\lambda }{H_{a}^{2}}\right)+\chi H_{a},$$

which describes the law of approach to saturation [60]. Parameter *M*_s_, as well as *δ* and *λ*, were set as free, while *χ* was fixed to zero, since for the considered samples, its effect was estimated to be negligible, due to the absence of paramagnetic features.

All the values of *M*_s_ are significantly lower than the one reported for Fe_3_O_4_ bulk material, which is around 480 kA/m [61]. This evidence can be related to a canting effect or a disorder of the magnetic spins at the surface of the Fe_3_O_4_ NPs, leading to a magnetic inactive (“dead”) layer that reduces the expected saturation magnetization [62,63]. The degree of this effect is basically determined by the surface-to-volume ratio, as confirmed by the fact that the value of *M*_s_ closest to the bulk one is found for sample #4, which contains the largest particles. Moreover, the hysteresis loops of samples #1 and #2 are characterized by a very slow approach to saturation with a marked non-saturating feature still at 1200 kA/m; whereas, a fully saturating behavior is observed in the loops of samples #3 and #4. The non-saturating effect observed in the first two samples could be ascribed to the fraction of MNPs with a size smaller than the critical one required to transit from superparamagnetism to ferromagnetism [59].

As reported in Table 1, the remanence to saturation ratio *M*_r_/*M*_s_ varies between 0.13 and 0.26. Sample #4 is characterized by the lowest value, due to the relative MNP size distribution, which comprises a predominant fraction of MNPs for which a multi-domain behavior is expected [59]. Among the first three samples, which have sizes more typical of the single-domain behavior, the lowest value of *M*_r_/*M*_s_ is found for sample #2, which contains the largest fraction of MNPs in the superparamagnetic state.

The magnetic domain configuration also affects the values of *H*_c_ (see Table 1), because of a different magnetization reversal process. The inversion of magnetization in single-domain MNPs (samples #1–3) results in a coherent rotation of the magnetization against the effective anisotropy properties. In multi-domain MNPs (sample #4) the magnetization reversal is characterized by a non-coherent rotation governed by the magnetostatic energy minimization, which is a magnetically easy process and consequently results in a smaller coercivity [61]. The variation in the values of *H*_c_ reflects the trend observed for the specific energy losses, estimated as

(12) $$E=\mu_{0}\oint H_{a}\cdot dM.$$

### 3.3. Analysis of the Role of Size and Effective Anisotropy via Micromagnetic Modelling

**Figure 4:**
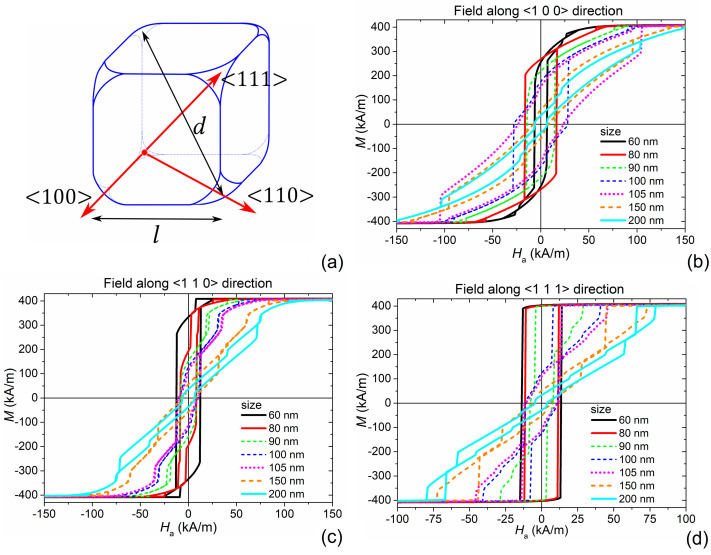
(**a**) Schematic of the cubic Fe_3_O_4_ NP with size *l*; the red arrows identify the three magnetocrystalline anisotropy axes, parallel to cube edge (<100> direction), cube face diagonal (<110> direction) and cube diagonal (<111> direction). Static hysteresis loops for a single Fe_3_O_4_ nanocube with *l* ranging from 60 nm to 200 nm, calculated by applying the magnetic field along (**b**) <100>, (**c**) <110> and (**d**) <111> directions.

To support the experimental magnetic characterization, we calculated the hysteresis loops of the Fe_3_O_4_ NPs by using the micromagnetic code described in Section 2.5, separately investigating all the factors that contribute to hysteresis losses. According to TEM analysis, in the simulations, the MNPs were approximated as truncated cubes with sizes ranging from 20 nm to 200 nm (between 10 nm and 20 nm transition from superparamagnetism to ferromagnetism is expected). Rounded corners were introduced, modelling the MNPs as objects obtained by intersecting a cube of side *l* with a sphere of diameter *d* = *l*(1 + √3)/2, being *d* the average between *l* and the cube diagonal length (schematic in Figure 4a).

First, we studied the influence of size on the hysteresis loop shape considering a single nanocube, i.e., disregarding the effects of magnetostatic interactions among MNPs. Thermal effects were not included in the simulations, after having initially verified their practically negligible contribution. Assuming that the crystal structure is aligned with the macroscopic structure of the cubic MNP (schematic in Figure 4a), the magnetocrystalline anisotropy easy and hard axes are oriented along the cube diagonals and the cube edges, corresponding to <111> and <100> directions, respectively. The cube face diagonals, <110>, are medium-hard axes. From a preliminary analysis, in which magnetocrystalline anisotropy was disregarded, the shape anisotropy, whose effect appears for size *l* larger than 80 nm, is characterized by easy axes along the <100> directions and hard axes along the <111> directions. Therefore, the effective anisotropy is a balance between the opposite magnetocrystalline and shape contributions.

**Figure 5:**
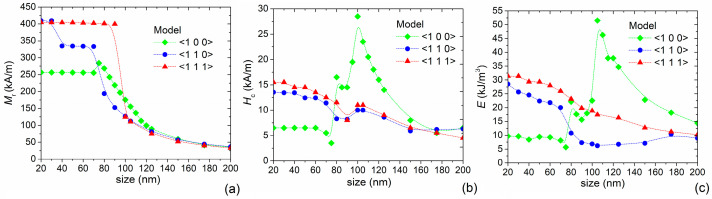
Influence of size (20–200 nm) on (**a**) remanent magnetization *M*_r_, (**b**) coercivity *H*_c_ and (**c**) specific energy losses *E* for Fe_3_O_4_ nanocubes, extracted from the static hysteresis loops calculated by applying the magnetic field along the <100>, <110> and <111> directions. The data are fitted with basis spline functions (dotted lines).

Figure 4 reports the static hysteresis loops calculated by applying the magnetic field along the three relevant directions for the effective anisotropy, for MNP sizes selected within the considered range of variation. The main properties of the computed hysteresis loops (remanent magnetization *M*_r_, coercivity *H*_c_ and specific energy losses *E*) are resumed in Figure 5, as a function of size.

When 20 ≤ *l* < 80 nm, small variations were found in the loop shape and size. In particular, a single-domain behavior was observed, with the effective hard axis along the cube edge or <100> direction (Figure 4b) and an effective easy axis along the cube diagonal or <111> direction (Figure 4d), due to the dominance of magnetocrystalline anisotropy effects. For the <100> direction *M*_r_ is around 60% of *M*_s_ (Figure 5a) and the values of *H*_c_ are in the order of 6.5 kA/m (Figure 5b). For *l* ≥ 80 nm, the hysteresis loop becomes wider, with a strong increase in coercivity, which reaches the peak value when *l* = 105 nm and then gradually reduces, arriving again at 6.5 kA/m for *l* = 200 nm. The loop area and thus the specific energy losses *E* (Figure 5c) reflect the non-monotonic behavior of coercivity, with a peak in the order of 50 kJ/m^3^ around 105 nm, confirming the trend observed by Li et al. [59]. This behavior, noticeable when the magnetic field is applied along the <100> direction, is due to the transition from a single-domain magnetic configuration to a multi-domain one.

Focusing on the hysteresis loops calculated along the <111> direction (Figure 4d), up to 90 nm, the remanent magnetization is close to saturation (Figure 5a), and for 20 ≤ *l* < 80 nm, the coercivity is strongly higher than the one found for the <100> direction, with a maximum of 15.5 kA/m for *l* = 20 nm (Figure 5b). Consequently, large specific energy losses were obtained (Figure 5c), in the order of 30 kJ/m^3^ for the smaller particles. A similar behavior, but with reduced values of *M*_r_, *H*_c_ and *E*, was observed when the magnetic field is applied along the <110> direction (Figure 4b). For the MNPs with *l* ≥ 80 nm (multi-domain regime), a decrease in *M*_r_, *H*_c_ and *E* occurs, more pronounced in correspondence of the transition sizes and for the <110> direction, as a consequence of the balance between shape and magnetocrystalline anisotropy effects.

### 3.4. Elucidation of Magnetization Reversal Process

**Figure 6:**
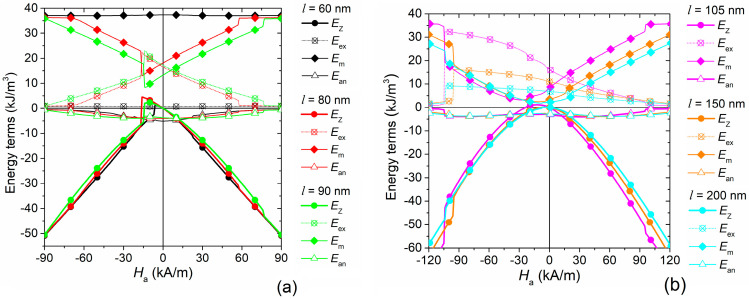
Evolution of energy terms versus applied magnetic field during the magnetization reversal process (along static hysteresis loop) of a single Fe_3_O_4_ nanocube with size *l* equal to (**a**) 60, 80 and 90 nm and (**b**) 105, 150 and 200 nm. In the legend *E*_Z_ stands for Zeeman energy, *E*_ex_ for exchange energy, *E*_m_ for magnetostatic energy and *E*_an_ for anisotropy energy.

**Figure 7:**
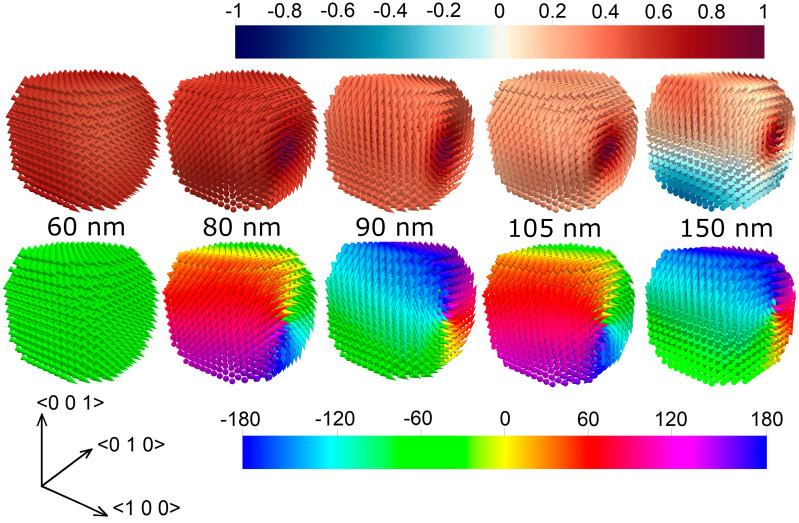
Remanence magnetization configuration for Fe_3_O_4_ NPs of different sizes, extracted from the static hysteresis loop calculated with the magnetic field applied along the <100> direction; the cones represent the magnetization orientation. The color bars refer to the normalized component of **M** along the <100> direction (top row) and to the angle (in degrees) between the <010> axis and the projection of **M** on the plane orthogonal to the <100> axis (bottom row). The top row well elucidates the decrease in *M*_r_ with size (see Figure 5a for the relative data).

The behavior observed when the magnetic field is applied along the <100> direction can be explained by analyzing the evolution, along the magnetization reversal process, of the different energy contributions versus size *l* (see Figure 6a for *l* = 60, 80 and 90 nm and Figure 6b for *l* = 105, 150 and 200 nm). When *l* ≤ 60 nm, the system tends to minimize the exchange energy, which remains very low for the whole process at the expense of the magnetostatic energy, being practically constant during all the reversal. When the applied magnetic field decreases, the magnetization gradually and coherently aligns to the easy axis, as confirmed by the reduction in the magnetocrystalline anisotropy energy and by the magnetization configuration at remanence shown in Figure 7. When the applied magnetic field increases in the opposite direction, the Zeeman energy rises until the magnetization overcomes the energy potential and coherently flips, realigning almost parallel to the easy axis, but with a change of sign of the magnetization component parallel to the field. From here, as the applied magnetic field increases, the magnetization reversibly moves from the easy axis to the field direction. For sizes *l* ≥ 80 nm, as the applied magnetic field reduces, the system starts to favor the minimization of the magnetostatic energy instead of the exchange energy. In a small central cylindrical volume, which connects the opposing faces perpendicular to the applied magnetic field, the magnetization is parallel to the field itself [64]. In the remaining part, the magnetization attempts to arrange itself in a closed path to minimize magnetic poles, following as much as possible the magnetocrystalline easy and medium axes directions with a vortex-like configuration (Figure 7).

For MNPs of medium size (*l* = 80–120 nm), when the applied magnetic field reaches coercivity, the magnetization in the central cylinder flips its orientation, while in the external part, the magnetization switches to a mirrored vortex configuration, where the component of the magnetization parallel to the field changes its sign. For larger sizes (e.g., 150 nm and 200 nm), the magnetization in the outer part follows the change of the applied magnetic field more gradually, while in the internal magnetic core, it goes through an irreversible jump at very high fields, as a consequence of its increase in length and thus in stability. This results in more slanted hysteresis loops, as illustrated in Figure 4b.

### 3.5. Analysis of the Role of Aggregation State via Micromagnetic Modelling

Sample #4, with a mean size *µ* of 162 nm, shows values of *M*_r_, *H*_c_ and *E* similar to the ones obtainable from micromagnetic modelling, by averaging the contributions of size dispersion (Figure 2) and randomness of the orientation of the MNPs with respect to the applied magnetic field (Figure 5). Within an interval around *µ* = 162 nm, the simulations are also able to predict a smooth magnetization reversal, as happens experimentally.

Stronger discrepancies between the measured and calculated properties were observed for the smaller samples (#1–3), specifically for the loop shape and *M*_r_, whose experimental value results to be in the order of 100 kA/m, i.e., highly lower than the ones computed by applying the magnetic field along the three relevant directions. A possible explanation to these differences can be provided by the presence of a non-negligible fraction of particles in the superparamagnetic state (Figure 2), as well as by the interparticle magnetostatic interactions, strongly influenced by the state of aggregation [31,35].

**Figure 8:**
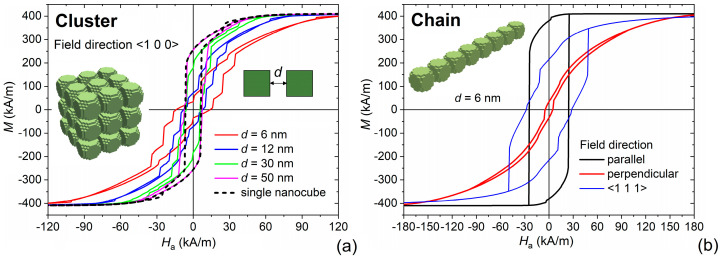
(**a**) Static hysteresis loops calculated for a cluster of Fe_3_O_4_ nanocubes with size *l* = 20 nm, arranged in a 3 × 3 × 3 grid, as a function of the mutual face-to-face distance *d*. The magnetic field is applied along the <100> direction. (**b**) Static hysteresis loops calculated for a chain of 8 nanocubes with *l* = 20 nm and *d* = 6 nm, as a function of the applied magnetic field direction.

Considering the higher level of aggregation of samples #1–3, deducible from TEM images in Figure 2, we calculated the static hysteresis loops of clusters made of 3 × 3 × 3 MNPs, with size of 20 nm and different face-to-face distance *d*, varied between 6 nm and 50 nm. The loops computed by applying the magnetic field along the direction <100> are reported in Figure 8a. In comparison to the case of single MNP, we observed a reduction in *M*_r,_ which reaches values in agreement with the measured ones when *d* = 6 nm; for reciprocal distances higher than 30 nm, the mutual interaction decreases significantly, and above 50 nm, it becomes almost negligible. Moreover, the loop shape is more similar to the experimental one, with a more gradual reversal, as a consequence of the non-synchronous switching of the magnetization, driven by the differences in the effective magnetic field acting on each MNP. According to the simulations, the magnetostatic interactions occurring in a cluster of MNPs are also responsible for an increase in coercivity, which practically doubles when *d* = 6 nm, approaching the experimental values for the direction <100>, too.

Chain arrangements can also be present, and they can lead to great values of *H*_c_ and *E* when the magnetic field is applied along the <111> direction or parallel to the chain, which corresponds to the easy axis for shape anisotropy. This is shown in Figure 8b, for a chain of 8 MNPs with size *l* = 20 nm and face-to-face distance *d* = 6 nm.

In conclusion, the overall properties of the analyzed samples can be numerically reconstructed by introducing an average of the different behaviors obtained by varying size, orientation of the MNPs with respect to the applied magnetic field and state of MNP aggregation (e.g., cluster, chain).

### 3.6. Heating Property Measurement and Thermal Modelling

**Figure 9:**
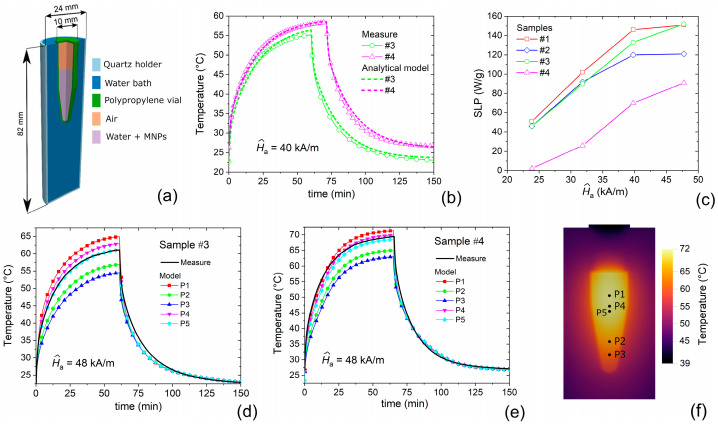
(**a**) Schematic of the sample container, corresponding to the domain considered for thermal modelling. (**b**) Time evolution of the temperature of the magnetic solution for samples #3 and #4, fixing the peak amplitude *Ĥ*_a_ of the alternating current (AC) magnetic field to 40 kA/m and its frequency to 100 kHz; the graph contains the experimental data and the best fit outputs of the thermodynamic analytical model [45]. (**c**) Comparison of measured SLP values for all samples as a function of *Ĥ*_a_. Comparison between measured and modelled heating-cooling transients for samples (**d**) #3 and (**e**) #4, considering *Ĥ*_a_ = 48 kA/m. (**f**) Spatial distribution of the temperature, calculated for sample #4 at the end of the heating interval (*t* = 65 min), with specification of the points where the temperature time-evolution in (**d**,**e**) was evaluated.

Thermometric measurements were conducted to evaluate the heating ability of all the samples (see the schematic of the sample container in Figure 9a). Figure 9b shows the time evolution of the temperature of the magnetic solution, measured for samples #3 and #4 under the application of an AC magnetic field with peak amplitude *Ĥ*_a_ = 40 kA/m and frequency *f* = 100 kHz. The fiber optic thermometer placed centrally in the magnetic solution records the temperature increase due to the MNP activation, when the AC magnetic field is switched on, and the temperature decrease, when the AC magnetic field is turned off and the solution is let cool down to room temperature again. The measured curves were fitted with a thermodynamic analytical model [45], developed to estimate the SLP values of MNPs.

The variation of the MNP heating efficacy as a function of the peak amplitude *Ĥ*_a_ of the AC magnetic field is depicted in Figure 9c for all the tested samples. The obtained values are in good agreement with the ones typically found for Fe_3_O_4_ NPs, which are spread in a wide range (10–200 W/g), in part due to the different AC magnetic field parameters (peak amplitude and frequency) used in the experiments [65,66]. Sample #4, containing the largest Fe_3_O_4_ NPs, shows the lowest SPL values. This suggests that the multi-domain magnetic configuration (sample #4) corresponds to a reduced power release (i.e., lower hysteresis losses) in comparison with the single-domain one (samples #1–3). The properties of the static hysteresis loops (*M*_r_, *H*_c_ and *E*), reported in Table 1, and the outcomes of micromagnetic modelling confirm this hypothesis. Samples #1–2 show an almost saturating behavior of SLP values above *Ĥ*_a_ = 40 kA/m, indicating that the dissipation mechanism has reached a full expression, i.e., the dynamic hysteresis loops are close to the major one. On the other hand, samples #3–4 show an evident rise of SLP values up to *Ĥ*_a_ = 48 kA/m; in this case, a further increase in the peak amplitude of the AC magnetic field may result in a further enhancement of the hysteresis losses and thus of SLP values [67]. However, the safety requirements for hyperthermia treatments [40] must be kept in mind for the selection of AC magnetic field parameters.

The experimental characterization was further corroborated by thermal simulations carried out with the numerical code described in Section 2.6, focusing on samples #3 and #4. The thermal modelling was performed on the system schematized in Figure 9a, which includes the vial containing the magnetic solution, where MNPs are uniformly dispersed, and the surrounding water bath. According to the experimental calibration process, we assumed a uniform coefficient of convective exchange *h* of 25 Wm^−2^K^−1^ between the holder surface and the external air. Table 2 resumes the materials properties used in the thermal simulations for the holder, made of quartz, the vial, made of polypropylene, the water bath and the air within the vial. In addition, it reports the effective parameters for samples #3 and #4, estimated by considering the fraction of MNPs dispersed in water [8,68].

The initial temperature of the system was assumed to be uniform in space and equal to the temperature of air *T*_ext_ in the surroundings of the sample holder. During the transient, *T*_ext_ in Equation (10) was approximated with a function that is uniform in space and variable in time. In particular, the time-behavior of *T*_ext_ was described with an exponential rise law and a subsequent exponential decay law during the heating and cooling phases, respectively. The two laws were obtained by fitting the curve of the temperature measured in the air region closer to the quartz holder wall. In this way, we took into account possible heating effects produced by the copper coil via Joule losses [69] and by the sample itself in the external air region. It is worth noting that at the end of the heating phase, an increase in *T*_ext_ up to 9 °C was found.

From the knowledge of the SLP values (Figure 9c) and MNP mass in the magnetic solution (5.43 mg for sample #3 and 9.82 mg for sample #4), *Q*_MNPs_ in Equation (9) was estimated to be 825 kW/m^3^ for sample #3 and 894 kW/m^3^ for sample #4, when the AC magnetic field has a peak amplitude *Ĥ*_a_ = 48 kA/m. The heating power *Q*_ext_ generated by the external field sources was estimated to be 45 kW/m^3^, as deduced from preliminary thermometric measurements without MNPs [45]. At the end of the heating phase, the heat sources were set at zero everywhere, enabling the system to cool down to room temperature. Figure 9d,e reports the heating–cooling transients calculated at different points within the magnetic solution for samples #3 and #4, respectively; the temperature gradient at the end of the heating phase is shown in Figure 9f for sample #4. As can be seen for points P4 and P5 (Figure 9f), which are the closest ones to the real position of the fiber optic thermometer, the simulation results are in good agreement with the experimental ones. This enables us to further confirm the validity of the thermodynamic analytical model [45] adopted for the calibration process and the estimation of the SLP values.

## 4. Conclusions

In this paper, we performed an extended study of Fe_3_O_4_ NPs, comprising preparation, characterization of their structural, morphological, dimensional, magnetic and heating properties, and micromagnetic and thermal modelling.

The hydrothermal method, chosen for the preparation, has proved to be a valid synthesis route for obtaining highly crystalline Fe_3_O_4_ NPs with well-defined cubic shape and average size in the range 15–160 nm, depending on temperature and reaction time. For the smaller samples, the size distribution reveals a large fraction of MNPs around the critical dimension for the transition from superparamagnetism to single-domain blocked state, while for the largest sample, the size range is typical of multi-domain configuration. As demonstrated by the magnetometric characterization, the magnetization reversal process is strictly influenced by the domain configuration; in fact, the remanence to saturation ratio and the coercivity are higher for single-domain MNPs than for the multi-domain ones. From thermometric measurements, performed in accordance with the safety requirements for hyperthermia treatments, the SLP values increase monotonically as a function of the magnetic field amplitude, but they are disadvantaged by the multi-domain configuration.

The experimental results were successfully supported by micromagnetic simulations, which have clarified the role of several factors in the generation of hysteresis losses, like MNP size, effective anisotropy (shape and crystalline contributions) and state of aggregation. The specific energy losses, calculated with the magnetic field applied along the cubic MNP edge, reflect the non-monotonic behavior of the coercivity, with a peak at 105 nm, i.e., after the transition from single-domain to multi-domain configuration, which occurs at 80 nm. Moreover, micromagnetic simulations allowed us to shed light on the magnetization reversal process, revealing a vortex-like configuration for the multi-domain MNPs.

Discrepancies between the calculated and measured magnetic properties were explained taking into account the wide distribution of size, orientation of the MNPs with respect to the applied magnetic field and state of aggregation in the analyzed samples. The high level of aggregation, observed in TEM images for the smaller samples, was taken into account by modeling MNP arrangement in chains and clusters. Depending on the orientation of the magnetic field, a smoother reversal process, more similar to the one observed experimentally, appears as a consequence of the non-synchronous switching of the magnetization in each MNP, impacting on the loop shape and thus on the specific energy losses.

## Figures and Tables

**Table 1:** Lattice constant and relevant magnetic properties for the four prepared samples. The values are affected by the coarse field sampling.

Sample	*a* (Å)	*M*_s_ (kA/m)	*M*_r_ (kA/m)	*M*_r_/*M*_s_	*H*_c_ (kA/m)	*E* (kJ/m^3^)
#1	8.3759	434.8	105.9	0.24	17.3	26.2
#2	8.3627	434.3	95.2	0.22	16.7	25.7
#3	8.3806	411.5	107.4	0.26	17.3	29
#4	8.3813	423.4	56.2	0.13	10.5	12.8

**Table 2:** Material properties used in the thermal simulations. The effective parameters for samples #3 and #4 were estimated according to [8,68].

Material	*ρ* (kg/m^3^)	*C*_p_ (J kg^−1^ K^−1^)	*k* (W m^−1^ K^−1^)
Water	997.05	4183	0.6
Quartz (holder)	2600	820	3
Polypropylene (vial)	905	1900	0.185
Air	1.16	1007	0.026
Sample #3	1019.8	4086	0.61
Sample #4	1038.1	4011	0.62

## Data Availability

The data presented in this study are openly available in Zenodo at DOI 10.5281/zenodo.5040394.
